# The effectiveness of delivery ball use versus conventional nursing care during delivery of primiparae

**DOI:** 10.12669/pjms.36.3.1440

**Published:** 2020

**Authors:** Jianfang Wang, Xuemei Lu, Chunhong Wang, Xuemei Li

**Affiliations:** 1 Jianfang Wang Public Health Section, Binzhou People’s Hospital, Binzhou, 256610, China; 2 Xuemei Lu Medical Records Room, Binzhou People’s Hospital, Binzhou, 256610, China; 3 Chunhong Wang Department of Obstetrics, Binzhou People’s Hospital, Binzhou, 256610, China; 4 Xuemei Li Department of Cardiovascular, Binzhou Shili Hospital, Binzhou, 256617, China

**Keywords:** Delivery, Delivery ball, Free position, Primipara

## Abstract

**Objective:**

To analyze the clinical effect of delivery ball and free position delivery nursing in primipara delivery.

**Methods:**

Total 110 primipara who were admitted to the department of gynecology and obstetrics of our hospital from August 2017 to August 2018 were included in the study. They were randomly divided into an observation group and a control group, 55 each group. The control group adopted conventional nursing measures, while the observation group adopted delivery ball combined with free position midwifery nursing on the basis of conventional nursing. The pain degree, comfort degree, pregnancy outcome and sense of delivery control were compared between the two groups.

**Results:**

The number of puerperae with grade 0 and 3 labor pain in the two groups had no statistically significant difference (P>0.05). The number of puerperae with grade 1 and 2 had significant difference (P<0.05). As to the comparison of the delivery mode between the two groups, there were 9 cases of cesarean delivery and 46 cases of vaginal delivery in the observation group; there were 19 cases of cesarean delivery and 36 cases of vaginal delivery in the control group; the differences had statistical significance (P<0.05). In the comparison of the birth process time of the vaginal delivery puerperae, the time of the first and second stages of labor in the observation group was shorter than that in the control group, and the differences were statistically significant (P<0.05); there was no difference in the time of the third stage of labor between the two groups (P>0.05). The amount of hemorrhage of the observation group 2 hours after labor was 172.50±40.60 mL and that of the control group was 224.45±32.80 mL; the difference between the two groups was statistically significant (P<0.05). The sense of delivery control of the puerperae who suffered vaginal delivery was compared between the two groups using Labor Agentry Scale (LAS); the sense of delivery control of the observation group was stronger than that of the control group, and the differences had statistical significance (P<0.05).

**Conclusion:**

In the delivery of primipara, delivery ball combined with free position delivery can help the primipara relieve pain, improve comfort degree, reduce the amount of postpartum hemorrhage, shorten the duration of various stages of labor, and improve the delivery outcome. It has a high clinical promotion value.

## INTRODUCTION

Delivery is a special physiological process of human beings. For women; pregnancy and delivery are both stressful events. In the process of delivery, women will have negative emotions, such as fear, anxiety, etc., especially primiparae.^1^,^2^ In addition, uterine contraction will aggravate maternal negative emotions, from psychological behavior to physical performance. If puerperae are excessively anxious and nervous, the increased release of catecholamine in the body will lead to hypertension and tachycardia and then induce uterine inertia, which will prolong the delivery; the severe consumption of strength may cause difficult labor, which can threaten the safety of mother and infants.^3^,^4^ Some puerperae choose cesarean section in order to avoid pain in the delivery process, but cesarean section is relatively unfavorable to the outcome of mothers and infants, and it is easy to increase the risk of postpartum hemorrhage; thus it is not advocated in clinic.^5^,^6^ Choosing the appropriate way to improve the natural delivery rate and improve the perception of the delivery process has become a clinical problem to be faced.

Free position delivery mainly refers to guiding puerperae to adopt comfortable position during delivery. Compared with traditional lying or semi-lying position, free position is more flexible and has no limitation on pelvic plasticity, which is conducive to promoting fetal delivery and alleviating labor pain.^7^ In recent years, delivery balls have been gradually used in obstetric delivery. As a rubber product, delivery balls have good flexibility and elasticity. They can massage the waist of puerperae, which is conducive to alleviating the psychological pressure and pain of the puerperal.^8^ A study points out that delivery ball and free position delivery play an important role in reducing maternal pain and the use of delivery ball and free position can effectively alleviate maternal pain during childbirth and improve comfort degree.^9^ In this study, 110 primiparae in our hospital were taken as the research subjects, and the effect of delivery ball combined with free position delivery nursing was observed. The purpose of this study was to further explore the application value of the scheme in the delivery process of primipara and provide a reference for clinical application.

## METHODS

### General data

From August 2017 to August 2018, 110 primiparae who were admitted to the department of obstetrics and gynecology in our hospital were selected as the research subjects.

***The inclusive criteria were:***

1. Primipara, without fertility experience
2. Single pregnancy confirmed by prenatal B-mode ultrasound
3. Full-term pregnancy
4. Having vaginal natural delivery indications
5. Being informed with research content and having signed informed consent.

Patients with pregnancy complications, mental disorders, and cognitive impairment, or severe heart, brain and blood diseases were excluded. This study was approved by the Ethics Committee of our hospital at the date of March 26, 2019. The primiparae were divided into a control group and an observation group, 55 each group, by random number table method. Primiparae in the control group was 24-37 years old (average (36.9±4.7) years) and had 38-41 gestational weeks (average (39.8±2.1) weeks). Primiparae in the observation group was 23-36 years old (average (36.8±4.6) years) and had 37-42 gestational weeks (average (38.5±1.7) weeks). There were no significant differences in the general data between the two groups (P>0.05); thus the results were comparable.

### Methods:

### The control group

The control group was given conventional nursing measures. The puerperae were delivered to the delivery room when the uterus was opened 3 cm and delivered in a conventional position (lying or semi-lying). In the delivery process, the midwifery nurses accompanied to pay close attention to the contraction of the uterus, guide breathing of the puerperae, and took effective measures in time if abnormal situation occurred.

### The observation group

On the basis of conventional nursing, the delivery ball and free position delivery nursing were used. The delivery ball was placed on a yoga mat, and handrails were set on the wall for the pregnant women to grasp. Nurses introduced the knowledge of delivery ball and free position to the pregnant women, instructed them to use delivery ball correctly, and helped them choose comfortable position to do free activities. The operation of the free position is as follows. When a standing position was used, the puerpera stood with the back against the wall and two hands grabbing the armrest. When a sitting position was used, the puerpera sat on the delivery ball with two hands grabbing the armest of the support and two feet stepping on the support and moved from side to side or upward and downward. When a kneeling position was used, the puerpera knelt on the mat, with the body forward, two hands holding the delivery ball and the head leaning on the ball, and waggled. When a squatting position was used, the puerpera was asked to squat besides the wall, and the ball kept close to the wall and the top of the ball was at same level of the shoulder blade. When a prone position was used, the delivery ball was placed on the delivery bed, the puerpera lay on the ball and shook her waist forward and backward or from side to side. In the whole midwifery process, the position and diameter of the delivery ball were adjusted according to the needs of the puerpera. The family members of the puerpera and nurses accompanied the puerpera in the whole delivery process, and moreover the puerpera were provided safety protection.

### Observation indicators:

1. The delivery pain was evaluated, grade 0 for no pain, grade 1 for mild pain, grade 2 for moderate pain and grade 3 for severe pain.^10^-^13^
2. The comfort degree was scored using General Comfort Questionnaire (GCQ). The total score was 28-112 points. The higher the comfort degree, the higher the score.
3. The anxiety and depression degrees were observed: Hamilton Anxiety Scale (HAMA) was used in the comparison of the anxiety condition between the two groups; the higher the score of HAMA, the severer the anxiety symptom. Hamilton Depression Scale (HAMD) was used in the comparison of the depression condition between the two groups; the higher the score of HAMD, the severer the depression symptom.
4. Observation of pregnancy outcome: The mode of delivery, the amount of postpartum hemorrhage in 2 hours and the time of the first, second and third stages of labor were observed.
5. Sense of delivery control: Labor Agentry Scale (LAS) which includes 29 items and 7 grades of score was used; the higher the LAS score, the better the sense of delivery control.^14^

### Statistical methods

SPSS 20.0 was used for statistical analysis of the data. The measurements were expressed as Mean±Standard deviation Mean±SD, and t test was used for analysis; X^2^ test was used in processing counting data. The difference was statistically significant if P<0.05.

## RESULTS

### Comparison of pain between the two groups

The difference of the number of puerperae with grade 0 and grade 3 delivery pain between the two groups had no statistical significance. There were 22 cases of grade 1 pain in the control group (40.0%) and 40 cases of grade 1 pain in the observation group (72.7%); the difference had statistical significance (P<0.05). There were 31 cases of grade 2 pain (56.4%) in the control group and 15 cases of grade 2 pain in the observation group (27.3%); there was statistical significance (P<0.05, Table-I).

**Table-I:** Comparison of pain between the two groups (%).

Group	Observation group	Control group	X^2^	P
Grade 0	0(0.0)	0(0.0)	/	/
Grade 1	40(72.7)	22(40.0)	7.829	<0.05
Grade 2	15(27.3)	31(56.4)	5.691	<0.05
Grade 3	0(0.0)	2(3.6)	0.714	>0.05

### Comparison of comfort degree between the two groups

The comfort degree score of the observation group was 103.49±17.21 during delivery, which was significantly higher than that of the control group (P<0.05, Table-II).

**Table-II:** Comparison of the comfort degree score between the two groups (Mean±SD, point).

Group	Comfort degree score
Observation group	103.49±17.21
Control group	85.38±14.65
t	5.554
P	<0.05

### Comparison of delivery modes between two groups

There were 9 cases of cesarean section and 46 cases of vaginal delivery in the observation group and 19 cases of cesarean section and 36 cases of vaginal delivery in the control group. The difference of delivery modes between the two groups was statistically significant (P<0.05, Table-III).

**Table-III:** Final mode of delivery between the two groups (%).

Group	Observation group	Control group	X^2^	P
Natural vaginal delivery	46(83.6)	36(65.5)	4.018	<0.05
Cesarean section	9(16.4)36(65.5)	19(34.5)		

### Comparison of postpartum hemorrhage volume and duration of labour between two groups

In the comparison of puerperae who delivered via vagina, the amount of postpartum haemorrhage within 2h in the observation group was significantly lower than that in the control group (P<0.05); the time of the first and second stages of labor in the observation group was shorter than that in the control group, and the difference was statistically significant (P<0.05); there was no significant difference in the time of the third stage of labour between the two groups (P>0.05, Table-IV).

**Table-IV:** Postpartum hemorrhage within 2 hand duration of labor between the two groups (Mean±SD).

Group	Observation group	Control group	t	P
Postpartum hemorrhage within 2h (mL)	172.50±40.60	224.45±32.80	5.308	<0.05
Time of the first stage of labor (min)	451.34±135.75	517.41±125.07	10.147	<0.05
Time of the second stage of labor (min)	30.80± 10.36	47.60±11.68	7.055	<0.05
Time of the third stage of labor (min)	5.52±1.46	6.17±2.53	0.675	>0.05

### Comparison of sense of delivery control between two groups

The sense of delivery pain of the puerperae who delivered through the vagina was compared between the two groups using LAS score. There were 36 puerperae who delivered through the vagina in the control group, and the LAS score was (123.61±17.22) points. There were 46 puerperae who delivered through the vagina in the observation group, and the LAS score was (169.38±18.72) points. There were statistically significant differences (t=7.659, P<0.05).

## DISCUSSION

Delivery is a physiological process most women experience. Uterine contraction is usually accompanied by obvious pain symptoms, which can result in poor maternal coordination and production of negative emotions such as anxiety and irritability, and it is the main cause for failure of delivery.^15^,^16^ Especially in traditional delivery, puerperae are required to maintain a fixed posture and guided by one nursing staff; the limited activities can aggravate the negative emotions of puerpera and prolong all stages of labor, including the first stage, the second stage of labor, etc., and some puerperae even strongly require the choice of cesarean delivery, which can affect the final outcome of delivery. Therefore, in order to reduce cesarean section and difficult labor, it is necessary to intervene pain of primipara during delivery.

At present, midwifery nursing is used for pregnant women during delivery. In the obstetric nursing mode, delivery ball and free position delivery nursing are new modes, and the frequency of their clinical application is increasing. As a delivery tool, delivery ball is designed for the majority of puerperae. The puerperae sit on the delivery ball, with their buttocks supported, the basin tissues are massaged through the spherical protrusion, the inclination of the pelvis can be improved, and the pressure of the lower limbs can be reduced; the sitting position can be maintained after uterine contraction, which can make the pelvic muscles relax.^17^,^18^ A study found that applying delivery ball in midwifery nursing could strengthen blood flow, reduce the muscle pressure, improve body coordination ability, and reduce energy consumption and it could also relieve pain and promote smooth delivery.^19^

In this study, the control group was given the traditional nursing, while the observation group was given delivery ball combined with free position delivery nursing on the basis of the control group. The research results demonstrated that the comfort degree score of the observation group was significantly higher than that of the control group, indicating that delivery ball in combination with free position delivery could help relieve the anxiety and improve the comfort degree, which was similar to the previous research results.^20^

The research results also demonstrated that the the vaginal delivery rate, the time of the first and second staged of labor and the amount of postpartum hemorrhage within 2 h in the observation group were all obviously superior to those of the control group, the pain of the observation group was weaker than that of the control group, and the LAS score of the observation group was higher than that of the control group. Xu et al. selected 210 puerperae for study.^21^ In their study, 105 puerpera who were given conventional midwifery mode was included into the control group, and the other 105 puerpera who were given midwifery mode combining delivery ball and free position were included into the observation group. It was found that the observation group was shorter labor time and higher natural delivery rate, and the incidence of postpartum hemorrhage, cervical laceration and vaginal wall laceration of the observation group was lower after delivery, reflecting that the effect of free position and delivery ball was remarkable, which was similar to the results of this study. The reason was that the puerperae sat on the delivery ball, with their buttocks supported, the basin tissues were massaged through the spherical protrusion, the inclination of the pelvis was improved, and the pressure of the lower limbs was reduced; the sitting position was maintained after uterine contraction, which can make the pelvic muscles relax. Moreover, the application of free position during the use of delivery could improve the comfort degree, distract attention, relieve pain and negative emotions, and enhance the sense of delivery control.^22^

## CONCLUSION

To sum up, the use of delivery ball and free position delivery during the delivery of primiparae has a positive effect on relieving anxiety and pain, and can help improve the outcome of delivery and reduce the risk of neonatal asphyxia, which is worth clinical promotion. However, this study is still insufficient as the sample size was too small, the follow-up time was too short, and the long-term effect of maternal delivery has not been analyzed. Thus further study with larger sample size needs to be carried out.

### Authors’ Contribution:

**JFW, XML, CHW & XML:** Study design, data collection and analysis.

**XML & XML:** Manuscript preparation, drafting and revising.

**JFW & CHW:** Review and final approval of manuscript.

**JFW** is responsible and accountable for the accuracy or integrity of the work.
